# High-Dose Remifentanil Anesthesia With Minimal Sedation in Neonates: A Single-Center Retrospective Study

**DOI:** 10.7759/cureus.67801

**Published:** 2024-08-26

**Authors:** Koshi Suwa, Kyosuke Takahashi, Yusuke Iizuka, Alan K Lefor

**Affiliations:** 1 Department of Anesthesiology and Critical Care Medicine, Jichi Medical University Saitama Medical Center, Saitama, JPN; 2 Department of Anesthesiology, Tokyo Medical and Dental University, Tokyo, JPN; 3 Department of Surgery, Jichi Medical University, Shimotsuke, JPN

**Keywords:** pediatric-anesthesia, remifentanil, neonate, general pediatric surgery, opioid

## Abstract

Introduction

Remifentanil is an opioid with rapid onset and elimination. Theoretically, reducing sedation using high-dose remifentanil may contribute to early emergence and prevention of postanesthetic complications related to residual anesthesia. However, there have been few reports of high-dose remifentanil anesthesia in neonatal surgery. This study aims to describe the techniques of high-dose remifentanil anesthesia in neonates and their safety outcomes.

Methods

This is a single-center, retrospective observational study from January 2016 to February 2022. Medical records from neonatal surgical procedures performed using high-dose remifentanil anesthesia were reviewed. “High dose” was defined as 0.5 mcg/kg/min or more. Patient profiles, anesthetic drugs used, and intra- and post-operative adverse events, including cardiopulmonary complications, were abstracted.

Results

There were 15 neonatal abdominal operations performed under high-dose (>0.5 mcg/kg/min) remifentanil anesthesia during the study period. The average remifentanil infusion rate was 1.9 (0.68-3.1) mcg/kg/min. Hypotension occurred in two patients (13%). Bradycardia was not observed in any patients. The mean time for tracheal extubation was 16 minutes. Five patients (33%) received naloxone administration before extubation, and two patients (13%) experienced hypoxemia immediately after extubation. No patient had cardiorespiratory complications after leaving the operating room.

Conclusions

High-dose remifentanil can be used without impairing hemodynamic stability in neonatal surgery, although there is concern about respiratory depression. Further research is needed on its potential impact on long-term outcomes.

## Introduction

Anesthesia for neonates requires specific considerations due to organ immaturity. Sensitivity to opioids in neonates is increased compared to older infants and adults, due to the limited development of the blood-brain barrier [1]. Hemodynamic instability and respiratory complications during and after anesthesia are common among neonates and necessitate careful titration of analgesics and sedatives. Remifentanil is an opioid with rapid onset and fast elimination, regardless of liver and kidney function; its half-time is 3.2 minutes, even after three hours of continuous infusion [2]. This pharmacological profile is preferable because the low accumulation of the drug could facilitate early emergence from anesthesia and prevent postanesthetic respiratory depression. Remifentanil also has sedative effects and is often used for procedural sedation in Intensive Care Unit settings [3,4].

Theoretically, reducing sedatives by using high-dose remifentanil has some possible advantages. First, we could expect fast recovery from anesthesia due to the characteristic of remifentanil's fast elimination, even in neonates. Sedatives and opioids, other than remifentanil, are metabolized by the liver and kidneys, making residual effects variable depending on organ function, particularly in newborns. Second, reducing sedatives may prevent potentially harmful effects on neurological development, such as apoptosis of neural cells in the immature rat brain, which some previous animal studies have shown [5].

Some reports regarding the use of remifentanil in neonates have demonstrated the utility of this drug for neonatal anesthesia [6-9]. Remifentanil is often used in combination with sedatives, and the dose of remifentanil varies across studies. Generally, remifentanil exhibits dose-dependent hemodynamic depression, with major adverse events being hypotension and bradycardia during general anesthesia [10]. There have been few reports on the use of high-dose remifentanil in neonatal surgery. Thus, the effects of high-dose remifentanil on hemodynamics and respiratory recovery remain unknown.

The present study conducted a retrospective analysis of neonatal surgical procedures performed using high-dose remifentanil anesthesia. This study aims to describe the anesthetic techniques and adverse events related to anesthesia.

## Materials and methods

This is a single-center, retrospective observational study. The Institutional Review Board of Jichi Medical University Saitama Medical Center approved the study design (Certification No. S22-047). The need for informed consent was waived due to the nature of this retrospective study. The authors obtained consent for publication by opt-out. Our study report adhered to the STROBE (Strengthening the Reporting of Observational Studies in Epidemiology) guidelines [11]. Medical records were reviewed for neonatal operations performed between January 2016 and February 2022. Data from patients treated with high-dose remifentanil were abstracted. “High dose” was defined as 0.5 mcg/kg/min or more. The dosage of remifentanil for neonates varies significantly across studies [6-9,12]; therefore, the choice of 0.5 mcg/kg/min was based on our clinical experience.

Patient characteristics and intraoperative data were obtained from hospital electronic medical records and anesthesia records. Patient characteristics abstracted included age, post-conceptual age, weight, gender, underlying disease, diagnosis, and surgical procedure performed. Intraoperative data included duration of anesthesia, operating time, time to tracheal extubation, anesthesia agents used, and vital signs during anesthesia. The time to tracheal extubation was defined as the time from the end of remifentanil infusion to tracheal extubation. Extracted vital sign data included minimum and average heart rate (HR) and blood pressure (BP) during surgery. BP was measured every two and a half to five minutes, and HR was recorded every minute. The following adverse events were collected: bradycardia (HR < 100/min), desaturation (SpO_2_ < 90%) [4], hypotension (mean BP < 30 mmHg) [13], laryngospasm, and use of naloxone.

The average infusion rate of remifentanil was calculated from the total remifentanil dose per kilogram during surgery, divided by the duration of surgery. Data are expressed as mean ± standard deviation (SD), or median with range, or number (percentage).

## Results

Patient profile

During the study period, 15 neonates underwent surgery under general anesthesia with high-dose remifentanil. A summary of patient characteristics is shown in Table 1. The mean age of the patients was seven days, and the mean weight was 2.9 kg. Three patients were late preterm infants, and two patients were low birth weight infants. Of these, one was a very low birth weight infant who had trisomy 18 with multiple organ abnormalities. Eleven patients had gastrointestinal surgery; of these, 10 patients underwent open laparotomy. Three patients had anal surgery, and one patient underwent an endoscopic procedure.

**Table 1:** Patient profile summary Data are expressed as mean ± standard deviation, median (range), or number (percentage). Remifentanil infusion rate, heart rate, blood pressure, and time to extubation are expressed as median (minimum-maximum value). The time to extubation is the duration between the end of remifentanil infusion and extubation.

Patient profile	Data
Age (days)	7 (1-30)
Post-conception age (weeks)	40 weeks, 6 days (35 weeks, 4 days-43 weeks, 1 day)
Weight (kg)	2.9 (1.4-3.8)
Gender (male)	10 (67%)
Surgical procedure	
Laparotomy (gastrointestinal surgery)	10 (67%)
Laparoscopic surgery	1 (6.7%)
Endoscopic surgery	1 (6.7%)
Others	3 (20%)
Operating time (min)	105 ± 38
Duration of anesthesia (min)	186 ± 52
Remifentanil infusion rate (mcg/kg/min)	1.9 (0.68-3.1)
Mean heart rate (/min)	171 (155-194)
Minimum heart rate (/min)	151 (130-167)
Mean blood pressure (mmHg)	48 (36-64)
Minimum blood pressure (mmHg)	36 (25-52)
Time to extubation (min)	16 (8-32)

Anesthetic management

Table 2 describes details of anesthesia and outcomes. All patients underwent tracheal intubation and mechanical ventilation during anesthesia. For 14 patients (93%), anesthesia was induced in the operating room and one patient had been intubated in the Neonatal Intensive Care Unit before being transferred to the operating room.

**Table 2:** Details of anesthetic drugs and outcomes FNT: Fentanyl; RMF: Remifentanil; PRP: Propofol; ROC: Rocuronium; MDZ: Midazolam; SEV: Sevoflurane; PHE: Phenylephrine; CaGlu: Calcium gluconate hydrate; EPH: Ephedrine The time to extubation is the duration between the end of remifentanil infusion and extubation. Desaturation is defined as an oxygen saturation of less than 90%.

Age (days)	Weight (kg)	Induction medications			Maintenance medications		Duration of anesthesia (min)	Time to extubation (min)	Desaturation in the operation room	Use of naloxone	Hypotension vasopressor
		Sedatives	Analgesics	Muscle relaxants	Infusion rate of remifentanil (mcg/kg/min)	Sedatives					
10	3.2	PRP 1.6 mg/kg	FNT 7.8 mcg/kg	ROC 2.8 mg/kg	2.3	-	186	32	-	-	-
22	3.7	PRP 1.6 mg/kg	-	ROC 1.1 mg/kg	2.5	SEV	254	8	-	-	-
29	3.0	PRP 2.0 mg/kg	-	ROC 1.0 mg/kg	1.0	-	174	13	-	0.01 mg	-
30	3.7	-	FNT 1.4 mcg/kg	ROC 1.0 mg/kg	2.6	SEV	285	Not extubated	-	-	-
2	2.8	-	FNT 3.6 mcg/kg	ROC 1.1 mg/kg	1.5	-	172	27	Yes	0.03 mg	-
27	2.8	PRP 2.1 mg/kg	FNT 3.6 mcg/kg	ROC 0.4 mg/kg	1.9	-	274	11	Yes	-	+nothing
7	2.7	-	RMF 3.8 mcg/kg	ROC 1.1 mg/kg	2.7	-	106	13	-	0.02 mg	−CaGlu
7	3.6	MDZ 0.1 mg/kg	FNT 4.2 mcg/kg	ROC 1.0 mg/kg	1.5	-	190	21	-	-	-
7	3.1	MDZ 0.2 mg/kg	FNT 3.2 mcg/kg	ROC 1.0 mg/kg	0.7	MDZ	176	16	-	-	-
1	3.8	-	FNT 3.9 mcg/kg	ROC 1.1 mg/kg	1.0	-	112	Not extubated	-	-	−CaGlu
13	1.4	-	FNT 7.1 mcg/kg	ROC 1.1 mg/kg	0.9	-	157	Not extubated	-	-	−DOA+DOB
3	2.7	PRP 1.1 mg/kg	-	ROC 1.9 mg/kg	2.5	-	195	8	-	0.01 mg	−CaGlu
1	2.8	-	-	ROC 1.1 mg/kg	3.1	-	124	Not extubated	-	-	+PHE+CaGlu
7	2.2	MDZ 0.2 mg/kg	FNT 4.7 mcg/kg	ROC 0.9 mg/kg	1.5	-	215	20	-	0.02 mg	-
3	2.9	-	FNT 1.7 mcg/kg	ROC 1.4 mg/kg	2.8	-	173	19	-	-	−EPH

Induction medications included propofol, midazolam, fentanyl, remifentanil, and rocuronium. For induction, rocuronium was administered to all patients. In five patients (33%), propofol was given for induction; three patients (20%) received midazolam for induction; whereas seven patients (47%) were induced without sedation. As analgesics, fentanyl was used in 10 patients (67%), and a bolus infusion of remifentanil was used in one patient (7%). The other four patients (26%) received a continuous infusion of high-dose remifentanil for anesthesia induction, without a bolus infusion of any drugs.

During the maintenance of anesthesia, the average remifentanil infusion rate was 1.9 (0.68-3.1) mcg/kg/min. Sevoflurane and midazolam were used for two (13%) patients and one (7%) patient, respectively. In the other 12 patients, anesthesia was maintained with high-dose remifentanil only, without sedatives. Eleven patients (73%) were extubated in the operating room, and sugammadex was used for all extubated patients. 

Adverse events

Hypotension was observed in two patients (13%), and bradycardia did not occur. During the operation, a few doses of vasopressors, such as phenylephrine, ephedrine, and calcium gluconate hydrate, were administered to five patients (33%). One patient required a continuous infusion of dopamine and dobutamine, which had started in the Neonatal Intensive Care Unit before being transferred to the operating room. Two patients (13%) had desaturation immediately after extubation. One patient recovered using continuous positive airway pressure delivered by a face mask, while one patient had laryngospasm, which required propofol administration to restore airway patency. Naloxone was administered to five patients (33%) before extubation. All patients were hemodynamically stable, without respiratory complications, after leaving the operating room.

## Discussion

The present study describes administering anesthesia using high-dose remifentanil with no or minimal sedation during 15 neonatal surgical procedures. Two patients experienced hypotension, but no bradycardia was observed in any patients. Two patients developed temporary hypoxemia after tracheal extubation.

Our results suggest that remifentanil itself may not necessarily induce hemodynamic instability. Since remifentanil is commonly used in conjunction with sedation, hypotension may be primarily caused by sedatives. A retrospective study described the use of remifentanil in 46 neonates undergoing non-cardiac surgery. The average infusion rate was 0.14 μg/kg/min, and most patients also received sevoflurane or other sedatives [8]. Another study focused on neonatal abdominal surgery found that an infusion rate of 0.25 μg/kg/min of remifentanil, combined with sevoflurane, was effective and well-tolerated in neonates [9]. Despite the modest doses of remifentanil used in previous studies, hypotension and bradycardia were reported as common complications [8,9,14]. In contrast, in the present study, hypotension and bradycardia were not frequent, and hemodynamic instability was not observed except in one patient, who required continuous infusion of catecholamines in the Neonatal Intensive Care Unit before remifentanil administration.

Hemodynamic instability during anesthesia using remifentanil, as described in previous reports, can be partially attributed to the use of sedation. One study reported hemodynamic changes after a bolus infusion of remifentanil, with stable BP and HR in early infant patients [15]. Additionally, two case reports described the anesthetic management of laparotomies for necrotizing enterocolitis in premature and very low birth weight newborns. The doses of remifentanil were high (Case 1: maximum dose 7.2 μg/kg/min; Case 2: maximum dose 3 μg/kg/min), along with a single bolus of midazolam, and bradycardia was not observed [12]. Considering that these cases did not experience hemodynamic compromise despite high-dose remifentanil infusion, the adverse effects on circulation could be induced by the concomitant use of sedation rather than by remifentanil itself.

Respiratory depression is one of the major side effects of opioid administration. Remifentanil is known to accumulate less than other opioids, due to its short context-sensitive half-time [16]. However, even though the half-life of remifentanil in early infants is approximately five minutes [16], postoperative apnea and hypoxemia after anesthesia using remifentanil have been reported [7,17]. A few patients in this study also developed hypoxemia after tracheal extubation, and 5 out of 11 extubated patients required the administration of naloxone before extubation. Although the use of naloxone may depend on the preferences of anesthesiologists, the percentage of naloxone use was quite high. Therefore, it should be noted that prolonged respiratory depression could occur after continuous infusion of high-dose remifentanil. Further investigations will be required to determine the dose relationships between remifentanil and the incidence of respiratory depression.

We hypothesized that high-dose remifentanil anesthesia would lead to a short time for tracheal extubation due to its rapid elimination. However, our results did not support the hypothesis. In this study, the time to tracheal extubation was 16 minutes, compared to 13 minutes in the previous study [8]. Additionally, interpreting the time to extubation in our study is complicated, as several influencing factors existed aside from remifentanil administration. First, the doses of fentanyl used in induction (4.1 mcg/kg) were higher than the typical dose (1-2 mcg/kg), which could have extended the time to tracheal extubation. Secondly, since naloxone was used in five cases, the time to tracheal extubation could have been significantly longer if it had not been used. Therefore, the effects of high-dose remifentanil anesthesia on time to extubation should be examined in prospective trials without these biases.

There are some concerns about high-dose remifentanil anesthesia. First, the combination of low-dose sedatives and high-dose remifentanil may predispose patients to intraoperative awareness. However, using high-dose remifentanil without additional sedatives may be justified, because remifentanil itself has a sedative effect. A previous study showed a dose-dependent decrease in values of the bispectral index (BIS) [13], suggesting that higher doses of remifentanil may provide sufficient depth of anesthesia for amnesia. Moreover, concerns about intraoperative awareness could be less critical in neonates than in older populations. According to cohort studies that estimated the incidence of accidental awareness during general anesthesia in children, the youngest patient was three years old [18]. However, we were unable to evaluate the sedative status in this study due to the lack of electroencephalographic monitoring during anesthesia.

Second, the effects of high-dose remifentanil on neurological development are controversial. An animal study has demonstrated that relatively high-dose remifentanil (80 mcg/kg/hr ≒ 1.3 mcg/kg/min) didn’t increase apoptosis caused by isoflurane in immature brains [19]. Based on these findings, remifentanil-centered anesthesia may not induce neurotoxicity in immature brains. However, there has been no research on the long-term effects of high-dose remifentanil in human neonates.

Finally, high-dose remifentanil may induce hyperalgesia. Some previous research showed that relatively high-dose remifentanil (0.6-0.9 mcg/kg/min) induced hyperalgesia in a dose-dependent manner [13]. On the other hand, a prospective randomized clinical trial reported the opposite phenomenon: high-dose remifentanil (1.2 mcg/kg/min) resulted in reduced postoperative visual analog scale (VAS) scores and morphine consumption compared to low-dose remifentanil (0.2 mcg/kg/min). It was also suggested that remifentanil, when administered beyond a certain dose, may paradoxically become less likely to induce hyperalgesia [20]. In this study, we used more remifentanil than in the previous studies and did not evaluate postoperative pain, so the incidence of hyperalgesia remains unknown.

To summarize, there is not enough evidence regarding the effects of high-dose remifentanil on intraoperative awareness, neurological development, and hyperalgesia in neonates. Further research on these topics is needed.

To the best of our knowledge, this is the largest case series to date demonstrating the feasibility of administering anesthesia using high-dose remifentanil with minimal sedation in neonatal patients. The present study does have acknowledged limitations. First, there is no comparison group because this is a retrospective descriptive study. Therefore, the superiority of clinical practice cannot be assured for safety and adverse events compared to conventional anesthesia. Second, the sample size was small, and the doses of remifentanil were highly varied because they were decided according to each anesthesiologist’s preference. Consequently, the findings of this study may lack sufficient replicability.

## Conclusions

In conclusion, high-dose remifentanil can be used without impairing hemodynamic stability in neonatal surgery. However, this technique raises concerns about respiratory complications. Further research is warranted to determine the effects of high-dose remifentanil on neonatal neurological development and hyperalgesia.
